# Understanding the roots of hesitancy to unlock the game-changing impact of HPV vaccination: Insights from a global qualitative evidence synthesis

**DOI:** 10.1080/21645515.2026.2681769

**Published:** 2026-06-23

**Authors:** Sara Cooper, Charles S. Wiysonge, Ngcwalisa A. Jama, Natalie Leon, Jill Ryan, Edison J. Mavundza, Rosemary J. Burnett, Asahngwa Constantine Tanywe, Dane Witten, Bey-Marrié Schmidt

**Affiliations:** a Cochrane South Africa, South African Medical Research Council, Cape Town, South Africa; b School of Public Health and Family Medicine, University of Cape Town, Cape Town, South Africa; c Department of Global Health, Stellenbosch University, Cape Town, South Africa; d Wits Health Consortium, University of the Witwatersrand, Johannesburg, South Africa; e Health Systems Research Unit, South African Medical Research Council, Cape Town, South Africa; f Equality Unit, Stellenbosch University, Stellenbosch, South Africa; g South African Vaccination and Immunisation Centre, Sefako Makgatho Health Sciences University, Pretoria, South Africa; h Department of Public Health Pharmacy and Management, Sefako Makgatho Health Sciences University, Pretoria, South Africa; i Cameroon Centre for Evidence-Based Healthcare, Yaounde, Cameroon

**Keywords:** Human papillomavirus (HPV) vaccination, vaccine hesitancy, qualitative evidence synthesis, cancer prevention

## Abstract

The human papillomavirus (HPV) vaccine is one of the most powerful tools in cancer prevention and is central to global efforts to eliminate cervical cancer as a public health problem. Yet worldwide uptake remains suboptimal. Our 2025 Cochrane qualitative evidence synthesis – comprising 71 studies from diverse geographic and socioeconomic settings – provides the most comprehensive global understanding to date of caregivers’ and adolescents’ views and practices regarding HPV vaccination, and the reasons why some are reluctant to receive the vaccine. Our review found HPV vaccine decision-making to be influenced by a complex web of socio-cultural, political and trust-related factors, beyond awareness or a simple evaluation of vaccine benefits and risks. We synthesized these factors into eight overarching themes. Together these provide a novel conceptual framework of the multi-level, interacting influencing factors, distinguishing it from more narrow, individual HPV vaccination acceptance frameworks. In this commentary we describe this conceptual framework and propose how it might be used to inform strategies for improving HPV vaccine uptake in ways that are more person-centered, equitable and ultimately effective. As countries worldwide aim to upscale and optimize HPV vaccination programs, incorporating insights from this review into HPV vaccination policy, program design and service delivery is essential and urgent.

Few vaccines have the potential to prevent cancer on the scale achieved by vaccines against human papillomavirus (HPV). HPV vaccination dramatically reduces HPV infection, precancerous lesions, and ultimately HPV-related cancer incidence.^1,2^ Its impact is further magnified by shifts to single-dose schedules and the introduction of gender-neutral programs in several countries.^3–5^

However, despite this public health promise, HPV vaccine coverage remains deeply uneven.^4,6^ Global coverage with the first dose of HPV vaccine among girls is estimated to be only 31%; far from the 90% target by 2030.^7^ While some high-income countries (HICs) have achieved high uptake, many low- and middle-income countries (LMICs) – which bear the highest burden of HPV-related cancers – continue to struggle.^8–10^ These gaps cannot be explained by supply constraints alone.^11^ They reflect complex behavioral, social, cultural, and logistical factors that determine whether caregivers and adolescents decide to accept HPV vaccination. Understanding these factors is therefore essential to realizing the vaccine’s full game-changing potential.

In 2025 we published a Cochrane qualitative evidence synthesis – based on robust, comprehensive literature searches conducted up to October 2024 – which explored the factors that influence caregivers’ and adolescents’ views and practices around HPV vaccination, and why some may be reluctant to receive the vaccine.^12^ The 71 studies included in the analysis came from all World Health Organization regions and included urban and rural settings, and high–middle–and low-income countries, creating a global spectrum of experiences. The synthesis offers a uniquely comprehensive view of how caregivers and adolescents think, feel, decide, and act in relation to HPV vaccination.

We synthesized the range of complex factors influencing caregivers’ and adolescents’ HPV vaccination views and practices into eight overarching themes namely: (1) Biomedical knowledge; (2) Perceptions of the risks and benefits (or lack thereof) of HPV vaccination; (3) Views and experiences of other vaccines and vaccination programs; (4) Nuclear familial decision-making dynamics; (5) Social networks, communities and the media; (6) Socio-cultural beliefs and practices; (7) Trust or distrust in the institutions, systems and experts associated with vaccination; (8) Access, supply and delivery logistics. Each theme represents a category of factors that may influence caregivers’ and adolescents’ views and practices regarding HPV vaccination. These themes are depicted graphically in Figure 1. Together these themes provide a novel and holistic conceptual framework of the multi-level, interacting influencing factors distinguishing it from more narrow, individual HPV vaccination acceptance frameworks.

**Figure 1.**
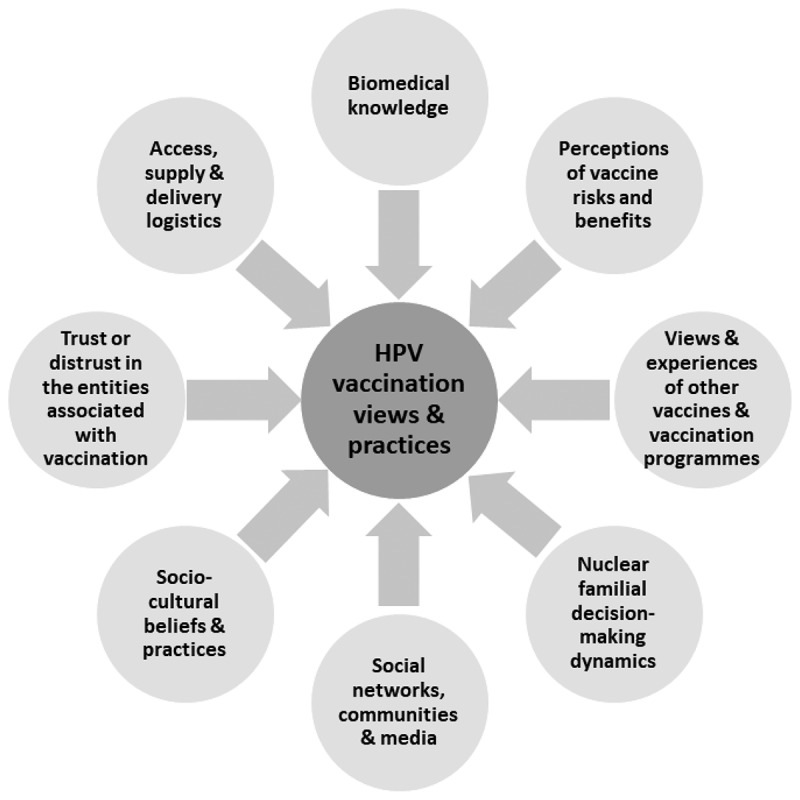
Conceptual framework: factors that influence caregivers’ and adolescents’ HPV vaccination views and practices.^12^

The first theme relates to persistent gaps in knowledge and awareness. Across studies, both caregivers and adolescents frequently demonstrated limited understanding of HPV, its link to cancer, or the preventive role of vaccination. Many were unaware that HPV is sexually transmitted, and some did not understand why vaccination is recommended at an early age before sexual debut. This widespread lack of knowledge about the vaccine influenced acceptance of it in complex and multiple ways. For some people it created a fertile ground for rumors, confusion, and hesitancy. For others it had no effect – they accepted the vaccine despite knowing very little about it. For certain people this lack of knowledge actually increased their acceptance of the vaccine. That is, they held erroneous beliefs about HPV vaccination from a biomedical perspective e.g. that it prevents pregnancy or protects against HIV/AIDS, yet these beliefs ironically served as strong motivators to receive the vaccine.

The second theme pertains to perceived risks, benefits and the “newness” of the HPV vaccines. Concerns about safety, side effects, and vaccine “newness” were prevalent across regions. Even in studies conducted more than a decade after introduction, many caregivers and adolescents viewed HPV vaccines as not yet sufficiently proven. Uncertainty about long-term effects, fear of adverse events, and questions about effectiveness contributed to people being conflicted about or opposed to HPV vaccination.

The third theme centered on caregivers’ and adolescents’ views and experiences of other vaccines and vaccination programs. That is, their acceptance of HPV vaccination (or not) had less to do with HPV vaccination itself, and was more about their relationship with other vaccines and vaccination more generally. For some, this relationship comprised a routine response to vaccination, and by extension, HPV vaccination. Others held particular opinions about, and previous experiences with, other vaccines and vaccination which shaped their HPV vaccination views and practices. For example, positive experiences with childhood vaccines often enhanced HPV vaccination acceptance. Conversely, negative experiences, such as perceived or real adverse events following other vaccinations or poor treatment in health facilities had a spillover effect, reducing trust in all vaccines, including HPV vaccines.

The fourth theme relates to nuclear familial decision-making dynamics, and the wide variation found in this regard and associated impact on HPV vaccination. In some settings, caregivers made decisions with little input from adolescents. In others, adolescents exercised considerable autonomy, without strong parental involvement. Sometimes caregivers felt that the decision should be made by adolescents because it is their body and sexuality, and thus delayed HPV vaccination until their adolescent was older and thought to be more equipped to make the decision themselves. In various contexts tensions emerged when adolescents and caregivers held opposing views, often leading to vaccine hesitancy or delays.

Wider social networks, communities and the media were also found to play a powerful influencing role, the fifth theme of the review. Parents and adolescents were often influenced by the vaccination decisions and opinions of those around them, including family members, peers, teachers, religious leaders, and local influencers. Many actively sought these entities for guidance or information. Social media stories (particularly those emphasizing risk or moral concerns) spread quickly and in some instances had a greater influence on HPV vaccine views and practices than formal health messaging.

The sixth theme involves cultural norms and moral values about adolescence, sexuality and gender. HPV vaccination often intersects with cultural beliefs about sexuality practices. Many caregivers feared that vaccination might encourage early sexual debut, multiple sexual partners, or complacency regarding safe sexual health practices. Several also associated HPV-related diseases with ‘immoral’ sexual behavior, such as promiscuity, and in turn viewed HPV vaccination as unnecessary or even shameful. These perceptions often overlapped with norms and values commonly attached to men and women, masculinity and femininity, leading to acceptance or refusal of HPV vaccination in gender differentiated ways. For example, many caregivers associated adolescent women with ideals of sexual innocence and virginity, as opposed to adolescent men that they commonly viewed as sexually curious and experimental. These social constructs of gender often led to decreasing acceptance of the HPV vaccine for women whilst increasing acceptance of it for men.

The seventh theme speaks to trust in institutions and experts. Trust or mistrust in government, pharmaceutical companies, schools, and healthcare workers played a fundamental role. The reasons for this trust or mistrust were diverse and often embedded within the intricacies of particular political events, relations, and processes within specific times and places. While the drivers of trust or mistrust thus varied, the impact was relatively consistent: high trust in the entities associated with vaccination contributed to enhancing acceptance whilst low trust contributed to magnifying uncertainty or fueling safety concerns.

The final theme focuses on vaccine access and delivery logistics, and the interplay between supply-related and demand-related dimensions of HPV vaccination. The accessibility, cost, and responsiveness of vaccination services strongly influenced whether parents and adolescents accepted the vaccine. For example, negative experiences–such as vaccine stock-outs or limited availability, high cost of the vaccine, poor communication and language barriers–often deterred parents and adolescents from accessing HPV vaccination, even if they believed in its benefits. In addition, practical and procedural aspects of vaccination delivery can have a potential impact on adolescents’ feelings toward the vaccine. For instance, in school-based vaccination programs, using privacy screens and distraction techniques or reducing the numbers of adolescents waiting together for vaccination was found to help reduce fears and the impact of peers’ negative reactions to vaccine administration.

Women-targeted HPV vaccination programs may also play a critical role. For some this ‘feminization’ has perpetuated a view of the HPV vaccine as a ‘female’ vaccine and therefore unnecessary or emasculating for men to receive. Others were suspicious or resented what they saw as the patriarchal norms of sexual and reproductive health being reinforced through female-focussed HPV programs – that sexual health is seen as a women’s responsibility. Others begrudged what they perceived as discrimination against men. Many supported gender-neutral vaccination as a more equitable approach, that might in turn enhance acceptance of the vaccine for all genders.

Taken together, these eight themes reveal how HPV vaccination acceptance (or not) is rarely a matter of information alone or a simple evaluation of the benefits and risks of the vaccine. Instead, it reflects deeper social, political, and moral contexts within which health decisions are made. Dominant conceptual models of vaccination decision-making generally^13,14^ and HPV vaccination specifically^12^ tend to focus on individual determinants and psychological processes. Our eight-theme conceptual framework extends these models by providing insights into the multiple factors and processes, across multiple levels influencing HPV vaccination views and practices. Our framework also reveals *how and why* different factors operate and overlap, further expanding dominant conceptual models which tend to focus predominantly on *what* factors influence vaccine acceptance.^15^ For example, it shows how individual risk perceptions about the vaccine encouraging early sexual debut or promiscuity interact with wider social norms and values about sexuality, adolescence and gender. Similarly, it reveals how broader political processes such as structural discrimination can produce distrust in authorities, which might spill over into the personal relationships people have with healthcare professionals and how they perceive the information or advice such professionals provide. All of these interrelated factors operating at various levels ultimately come together to reduce acceptance of HPV vaccination.

The findings from our review have several implications for policy and practice. The drivers of HPV vaccine hesitancy are multiple and often vary across place, time and population, as revealed by our review. Understanding and then targeting context specific drivers within particular settings and populations is therefore essential. No single and one-size-fits-all strategy is thus likely to be effective. Our eight-theme conceptual framework could serve as a basis for gaining this understanding and developing responses that are more aligned with the norms, values and concerns of caregivers and adolescents. It could also be used to inform which levels of influence (e.g. societal, institutional, interpersonal, intrapersonal) intervention strategies might need to target and potentially combine, if they are to be successful.

Most significantly, the review findings underscore the importance of shifting from information provision and awareness-raising to more socio-behaviorally informed communication. Here valuable insights can be drawn from risk communication sciences,^16–19^ particularly those that stress engagement, transparency, and trust-building. Knowledge alone is insufficient, and communication must also address emotions, social norms, and moral concerns. This necessitates genuinely listening to parents’ and adolescents’ concerns about the HPV vaccine, understanding the wider social norms within which these may reside, and considering how these might be addressed in meaningful and sensitive ways. For example, using gender-neutral communication approaches such as framing HPV vaccination as a cancer-prevention tool for all genders might help diminish the stigma and value judgments around sexuality so often imbued in representation of HPV vaccination.^20^ Deeper community-level work might also be necessary to help people question the negative ideologies about gender, sexuality and adolescence that have permeated understandings of the vaccine, and to legitimize alternatives. Here community engagement initiatives around HIV/AIDS that worked with communities to challenge the moral codes and othering tendencies shaping representations of HIV^21–25^ could be emulated and adapted for HPV vaccination.

Effective communication also requires building trust, potentially at multiple levels. Open and honest engagement, including communication on potential vaccine risks, side effects, knowledge gaps and uncertainties would be important here. It might also be helpful to identify which individuals or institutions are trusted – such as healthcare workers, teachers, civil society organizations, religious or traditional leaders – and potentially including these trusted entities in the design or implementation of programs. This could help to bring communities on board, foster local ownership and ultimately build social support for HPV vaccination. Understanding and targeting the deeper reasons for distrust in entities associated with vaccination may also be necessary. Here it could be beneficial to consider dialogue-based approaches which invite open discussion and potentially awkward conversations about politics, power, and interests, and broader structural injustices people may face.^26^ This could help communities feel more heard and be less distrusting, and in turn more willing to listen.

It is imperative for this kind of HPV communication to happen early on and often. A striking finding from the review was the limited change in vaccine views and experiences over time. The studies included in the review were published over a 15-y period (2008–2023) yet we found very little change in the core drivers of HPV vaccine hesitancy. This suggests deeply rooted concerns that require sustained, ongoing engagements over time, rather than one-off interactions.

The review findings also highlight the significance of addressing the structural and service-level issues that may be hindering the accessibility and quality of HPV vaccination services, and in turn reducing motivation to access these services. For instance, it is important to consider how HPV vaccination could be provided at more convenient locations, such as through school-based vaccination outreach or mobile vaccination teams. It is also important to explore whether gender-neutral HPV vaccination programs could be a feasible and affordable option in the target setting.

There should also be consideration for how adolescents might be better centered in the design of HPV vaccination programs. Other reviews suggest that adolescents’ HPV vaccination views and experiences are underrepresented in the literature.^27,28^ However, our review did not support this perspective – we identified over eighty studies that included adolescents only or both adolescents and caregivers as study participants. The issue may therefore be less about whether there is evidence on adolescents’ HPV vaccination views and experiences, and more about the need for this evidence (and these perspectives) to be better incorporated into immunization programs. For example, it could be worth considering how adolescents’ anxieties and peer influences could be directly addressed in scheduling, layout, and communication strategies for school-based and facility-based services.

In conclusion, HPV vaccination holds immense promise as a game-changer in the fight against HPV-associated cancers. Yet scientific breakthroughs can only translate into public health impact when they resonate with the lived experiences of those they intend to protect. Our Cochrane qualitative evidence synthesis on HPV vaccination provides the most globally comprehensive insight to date into these lived realities. Its eight themes offer a roadmap for designing HPV vaccination programs that are not only clinically effective but also socially resonant, culturally sensitive, and person-centered. Integrating these insights into policy and program design will be essential for achieving high HPV vaccine uptake, closing equity gaps, and accelerating global progress toward the elimination of cervical cancer and other HPV-associated cancers.
